# Time-Dependent Phospholipid Remodeling in Cultured Primary Mouse Hepatocytes: Associations with PEMT Status and Methionine Availability

**DOI:** 10.3390/cells15171608

**Published:** 2026-09-03

**Authors:** Tianxin Ma, Kunpeng Zhou, Yibing Zhu, Jiale Zhang, Xing Xie, Lu Zhang, Cunqi Ye, Zong-Cai Tu

**Affiliations:** 1 College of Chemistry and Materials, Jiangxi Normal University, Nanchang 330022, China; 18702515908@163.com; 2 Zhejiang Provincial Key Laboratory for Cancer Molecular Cell Biology, Life Sciences Institute, Zhejiang University, Hangzhou 310058, China; 3 Key Laboratory of Reproductive Genetics (Ministry of Education) and Department of Reproductive Endocrinology, Women’s Hospital, Zhejiang University School of Medicine, Hangzhou 310006, China; 0625184@zju.edu.cn; 4 College of Life Sciences, Natl R&D Branch Ctr Convent Freshwater Fish Proc, Jiangxi Normal University, Nanchang 330022, Chinazhanglu00104@163.com (L.Z.); 5 State Key Laboratory of Food Science and Resources, Nanchang University, Nanchang 330047, China

**Keywords:** primary mouse hepatocytes, membrane phospholipid remodeling, PC/PE balance, PEMT, methionine–SAM metabolic axis

## Abstract

**Highlights:**

**What are the main findings?**

- Culture time was the dominant factor associated with sequential membrane phospholipid remodeling in primary mouse hepatocytes within 0–72 h, with stage-specific changes in PC/PE balance, acyl chain length and lipid unsaturation.
- PEMT was associated with PC/PE composition without controlling hepatocyte dedifferentiation; methionine deprivation partially overlapped with the lipid profiles of PEMT knockout.

**What is the implication of the main finding?**

- Dynamic phospholipid characteristics, including the PC/PE ratio, acyl-chain length and unsaturation patterns, may serve as potential indicators of the culture-associated state of primary hepatocytes.
- This work links hepatocyte culture adaptation with phospholipid remodeling and methionine-related metabolic changes.

**Abstract:**

Background: Cultured primary mouse hepatocytes undergo drastic phenotypic and metabolic reprogramming, while the temporal rules and regulatory machinery of membrane phospholipid remodeling remain elusive. Methods: Relying on a 0–72 h time-series in vitro culture system, this study integrated multi-omics technologies to dissect the temporal dynamics of phospholipid remodeling in hepatocytes. Through phosphatidylethanolamine N-methyltransferase (PEMT) knockout, exogenous PEMT expression, and methionine deprivation, we examined the association of PEMT status and methionine availability with phospholipid remodeling. Results: In vitro cultivation reduces intracellular total phospholipids, phosphatidylcholine (PC) and phosphatidylethanolamine (PE) through three coordinated events: suppressed transcription of phospholipid synthetic genes hinders de novo synthesis, elevated lipid hydrolysis consumes cellular phospholipids, and extracellular phospholipids accumulate in the culture medium from 12 to 48 h. These jointly trigger ordered remodeling of PC/PE balance, acyl chain length and fatty acid unsaturation. PEMT knockout was associated with PE retention without worsening hepatocyte dedifferentiation, PEMT exogenous expression raises PC content and PC/PE ratio yet cannot rescue culture-dominated lipid structural shifts. Methionine depletion depleted cellular methionine, S-adenosylmethionine (SAM) and S-adenosyl-L-homocysteine (SAH), producing selected lipid changes that partially overlapped with lipid phenotypes of PEMT knockout. Conclusion: In short, culture duration was the dominant factor associated with the fundamental phospholipid remodeling trajectory, and PEMT status and methionine availability, were associated with selective differences in lipid composition.

## 1. Introduction

Primary hepatocytes are an important in vitro model for studying hepatic metabolism, as they retain, to some extent, the metabolic characteristics and functional state of hepatocytes [1]. They are therefore widely used to investigate lipid metabolism [2], drug metabolism [3], nutritional intervention and liver-disease modeling [3,4]. However, after transfer to two-dimensional culture, primary hepatocytes rapidly lose characteristics and specific functions, downregulate hepatocyte-specific functional genes [5], remodel metabolic programs [6] and show reduced albumin secretion [7], urea synthesis [8] and cytochrome P450 activity [4,6]. Existing strategies, including collagen sandwich culture, co-culture systems, extracellular matrix optimization and liver-on-a-chip platforms, mainly aim to delay dedifferentiation and preserve liver-specific functions [8,9]. Although these approaches have improved the maintenance of hepatocyte function in vitro, they also underscore that primary hepatocyte culture is not a static condition but a dynamic process of cellular state transition.

Hepatocytes are central regulators of lipid metabolism, supporting fatty acid synthesis, triglyceride handling, cholesterol metabolism, lipoprotein assembly and membrane lipid renewal [10]. Phospholipids are an important part of this metabolic network because they form the structural basis of plasma and organelle membranes and contribute to membrane composition, organelle function and metabolic compartmentalization [11,12]. Previous studies have shown that hepatocytes undergo marked alterations during isolation and in vitro culture, including changes in redox and energy metabolism, sphingolipid accumulation, reduced phosphatidylethanolamine (PE) and phosphatidylinositol (PI), increased ratio of saturated fatty acids (SFA) to monounsaturated fatty acids (MUFA), and decreased polyunsaturated fatty acids (PUFA) [6,13]. However, how membrane phospholipid composition changes over the early course of primary hepatocyte culture, and whether these changes are associated with specific phospholipid metabolic pathways, remain insufficiently understood.

Among membrane phospholipids, phosphatidylcholine (PC) and phosphatidylethanolamine (PE) are two major classes in mammalian cells [14]. Their relative abundance and molecular composition, including fatty acyl-chain length and unsaturation, contribute to membrane lipid organization and may reflect changes in cellular state [15,16,17]. PC and PE are mainly synthesized through the Kennedy pathway [18], in which the CDP-choline branch produces PC and the CDP-ethanolamine branch produces PE [19,20]. In hepatocytes, PE can also be converted to PC by phosphatidylethanolamine N-methyltransferase (PEMT), a liver-enriched enzyme that uses S-adenosylmethionine (SAM), derived from methionine metabolism, as the methyl donor and generates S-adenosyl-L-homocysteine (SAH) [21,22].

Primary hepatocytes rapidly undergo functional decline during in vitro culture, accompanied by extensive transcriptional and metabolic changes. Previous studies have observed alterations in lipid content and composition during culture. However, these changes are typically summarized as culture-induced lipidome shifts [13].

Studies systematically characterizing temporal membrane phospholipid remodeling in primary hepatocytes upon ex vivo culture are still lacking. It is unknown whether phospholipid remodeling proceeds along specific time-dependent trajectories during hepatocyte early adaptation and subsequent functional deterioration. In addition, how PC/PE homeostasis, phospholipid subtypes, acyl chain features and lipid unsaturation vary over these stages also awaits comprehensive exploration. PEMT catalyzes the conversion of PE to PC using SAM as a methyl donor, producing SAH in the process, thereby linking phospholipid synthesis to methionine metabolism. The biochemical functions and substrate preferences of PEMT have been thoroughly documented by previous studies. Nevertheless, how PEMT expression/activity shifts throughout primary hepatocyte culture, as well as whether such alterations modulate the time-dependent dynamics of PC/PE balance and phospholipid molecular structure, still requires clarification. Furthermore, it remains to be verified whether culture-induced phospholipid remodeling is coupled to the methionine–SAM–SAH metabolic axis. In this study, we conducted continuous observations of primary mouse hepatocytes over 0–72 h and combined these with PEMT knockout, PEMT overexpression, and methionine deprivation interventions to analyze the dynamic changes in membrane phospholipids in terms of lipid classes, molecular species, acyl chain length, unsaturation, and methyl donor metabolism. We focus on three interrelated questions: how PC and PE are remodeled during primary hepatocyte culture; whether altering PEMT levels can redirect or partially restore this remodeling trajectory; and whether this process is accompanied by changes in the methionine–SAM–SAH metabolic axis. Through these analyses, this study aims to elucidate the role of PEMT in culture-induced membrane phospholipid remodeling, and to provide a basis for identifying time-dependent lipid signatures that reflect the state of primary hepatocyte culture.

## 2. Materials and Methods

### 2.1. Materials

All reagents used for HPLC analysis were HPLC grade, methanol (Optima™ LC/MS grade; Cat. no. A456-4; Fisher Chemical, Thermo Fisher Scientific, Waltham, MA, USA), acetonitrile (HPLC grade; Cat. no. AC610010040; Thermo Scientific Chemicals, Thermo Fisher Scientific, Waltham, MA, USA), chloroform (HPLC Plus; Cat. no. 650498; Sigma-Aldrich, St. Louis, MO, USA). SETA (NSK-A; Cambridge Isotope Laboratories, Inc., Tewksbury, MA, USA), lipidomix, PC (17:0), PE (17:0), and PS (14:0) were obtained from Avanti (Avanti Polar Lipids, Alabaster, AL, USA). All other reagents used were analytical grade. Insulin-transferrin-selenium (ITS-G, 100×; Cat. no. 41400045) and William’s E medium (Cat. no. 12551032) were purchased from Gibco, Thermo Fisher Scientific, Grand Island, NY, USA. Dexamethasone (at. no. D4902; CAS no. 50-02-2) and Collagenase type IV from Clostridium histolyticum (Cat. no. C5138; CAS no. 9001-12-1) were purchased from Sigma-Aldrich, St. Louis, MO, USA. Collagen type I, rat tail (Cat. no. 354236), was purchased from Corning Life Sciences, Tewksbury, MA, USA. EGTA (Cat. no. 324626; CAS no. 67-42-5) was obtained from Millipore (EMD Millipore Corporation, Burlington, MA, USA). Hanks’ Balanced Salt Solution (HBSS; Cat. no. C0218) was purchased from Beyotime Biotechnology, Shanghai, China. Penicillin-streptomycin solution (100×; Cat. no. E607011) was purchased from BBI (Sangon Biotech (Shanghai) Co., Ltd., Shanghai, China).

### 2.2. Animals

Wild-type C57BL/6 male mice (6–8 weeks) were purchased from GemPharmatech Co., Ltd. (Nanjing, Jiangsu, China). *Pemt^−/−^* mice were kindly provided by Prof. Cunqi Ye (Zhejiang University), and genotyping was performed according to previously described protocol [23]. To establish a mouse model of PEMT overexpression in the liver, the mouse PEMT coding sequence (*Pemt*, NM_008819.3) was cloned into an AAV vector and driven by a liver-specific TBG promoter. The recombinant virus was AAV8 serotype, and was packaged and purified by Jike Gene (Shanghai GeneChem Co., Ltd., Shanghai, China.). Experimental group: TBG-Pemt-3Flag-FT2A-EGFP-WPRE-BGHpolyA, control group: TBG-3Flag-FT2A-EGFP-WPRE-BGHpolyA, with a viral titer of ≥1 × 10^12^ v.g./mL. After 1 week of acclimatization, WT mice were administered the viral solution via portal vein injection (1.5 × 10^11^ v.g. per mouse). Following anesthesia, the abdomen was opened to expose the portal vein, and 250 μL of the viral solution diluted in PBS was injected using an insulin syringe. The incision was then sutured and the abdomen closed. After recovery, the mice were maintained under standard conditions for 4–6 weeks. All mice were housed in pathogen-free conditions at 22–24 °C with a 12 h light/dark cycle. The animal study protocol was approved by the Institutional Review Board of Animal Care and Use Committee of Zhejiang University (protocol code: ZJU20230437; date of approval: 7 November 2023).

### 2.3. Isolation and Culture of Primary Mouse Hepatocytes

Primary mouse hepatocytes were isolated using a modified two-step collagenase perfusion method [24]. Briefly, 8-week-old male mice were anesthetized and the inferior vena cava was cannulated to allow for retrograde liver perfusion. The liver was sequentially perfused at a flow rate of 6 mL/min with 50 mL of HBSS containing 2.5 mM EGTA, followed by 25 mL of HBSS supplemented with 2.5 mM CaCl_2_ and 0.05% collagenase type IV from Clostridium histolyticum. All solutions were pre-warmed to 37 °C. Post-perfusion, the liver lobes were collected and transferred into a 100 mm dish containing cold plating media (William’s E medium with 10% FBS (ExCell Bio, Suzhou ExCell Biotechnology Co., Ltd., Suzhou, China), 1× ITS, and 40 ng/mL dexamethasone, and 1% penicillin-streptomycin). Hepatocytes were released by gentle agitation, filtered through a 100 μm cell strainer (Corning), and washed three times with cold plating media via centrifugation, followed by low-speed centrifugation and purification using 60% Percoll. The viability of the purified hepatocytes was assessed by trypan blue exclusion using a hemocytometer. To minimize variability in cell quality, only preparations with a viability above 80% were used for plating and subsequent experiments. Viable hepatocytes were seeded onto collagen-coated plates (pre-incubated with 50 μg/mL collagen for 6 h, rinsed with 1× PBS, and dried) with plating medium (William’s E medium with 10% FBS, 1× ITS, and 40 ng/mL dexamethasone, and 1% penicillin-streptomycin) at a density of 2 × 10^5^ cells per well. For each independent preparation, hepatocytes isolated from 2 mice were pooled before plating. Four wells were seeded from each pooled cell suspension for each experimental condition. Freshly isolated and purified primary mouse hepatocytes collected immediately before plating were defined as the 0 h samples. The remaining cells were seeded onto plates and collected after 4, 12, 24, 48, and 72 h of culture. After 4 h of attachment, the plating medium (William’s E medium with 10% FBS, 1× ITS, and 40 ng/mL dexamethasone, and 1% penicillin-streptomycin) was replaced with incubation medium (William’s E medium containing 1× ITS, 40 ng/mL dexamethasone, and 1% penicillin-streptomycin). The medium was changed once at 48 h. At each medium change, the culture medium was removed, and the cells were gently washed with pre-warmed PBS before fresh incubation medium was added.

### 2.4. Collection, Extraction and LC-MS/MS Analysis of Cellular Metabolites from Primary Mouse Hepatocytes

After removal of the culture medium, cells were washed twice with PBS and quenched with liquid nitrogen. Then, 1 mL of pre-cooled 60% LC-MS-grade methanol was added to each well, and cells were scraped and transferred into 1.5 mL screw-cap tubes. After snap-freezing in liquid nitrogen, samples were subjected to five freeze–thaw cycles with shaking at room temperature (67 Hz, 180 s). Samples were then centrifuged at 15,000× *g* for 10 min at 4 °C. A total of 800 μL of the supernatant was collected and centrifuged again. Subsequently, 700 μL of the supernatant was transferred into new EP tubes in two aliquots and completely dried using a vacuum concentrator. The dried samples were stored at −80 °C until further analysis.

For LC-MS/MS analysis, dried samples were reconstituted in 120 μL of 60% acetonitrile/H_2_O, followed by addition of the SETA internal standard. Samples were shaken at 20 Hz for 10 min and then incubated on ice for 30 min. After centrifugation at 15,000× *g* for 10 min at 4 °C, the samples were centrifuged twice, and 80 μL of the supernatant was transferred into LC-MS sample vials.

Cellular metabolites were quantitatively analyzed by LC-MS/MS using a triple quadrupole mass spectrometer (QTRAP 5500+ system, AB SCIEX LLC, Marlborough, MA, USA) according to an in-house established method. Briefly, metabolites were separated on a SeQuant ZIC-pHILIC column (5 μm polymer, 150 mm × 2.1 mm; Millipore Sigma) using a high-performance UHPLC system (ExionLC AD system) coupled to a triple quadrupole mass spectrometer (QTRAP 5500+ system, AB SCIEX). For targeted metabolomics, chromatographic separation was performed on the HILIC column over 34 min at a flow rate of 0.15 mL/min. Solvent A consisted of 20 mM ammonium carbonate and 0.1% (*v*/*v*) ammonium hydroxide, and solvent B was acetonitrile. The gradient was as follows: 0.01 min, 80% B; 20 min, 20% B; 20.5 min, 80% B; and 34 min, 80% B. Raw data were extracted using Analyst software v1.7.2 and OS v1.7.

### 2.5. Collection, Extraction and LC-MS/MS Analysis of Lipids from Primary Mouse Hepatocytes

After removal of the culture medium, cells were washed twice with PBS and quenched with liquid nitrogen. Then, 500 μL of 80% LC-MS-grade methanol was added to each well, and cells were scraped and transferred into tubes. The collected cells were subjected to five freeze–thaw cycles with shaking at 67 Hz for 180 s. The samples were then transferred into glass tubes and mixed with 1 mL of chloroform, vortexed for 30 s, and centrifuged at 1000× *g* for 10 min at room temperature. The supernatant was collected and mixed with 400 μL of chloroform and 200 μL of 50 mM citric acid. After vortexing for 30 s, the samples were centrifuged again at 1000× *g* for 10 min at room temperature. The liquid phase was collected and dried using a vacuum concentrator. A defined spike-in standard mixture containing PC (17:0), PE (17:0), and PS (14:0) was added to each sample during lipid extraction to monitor extraction recovery. For medium lipid extraction, collected culture medium was centrifuged at 15,000× *g* for 10 min to remove suspended cells and cellular debris. A 200 μL aliquot of the clarified supernatant was dried and extracted using the same lipid extraction procedure as described for cell samples. Before drying, each sample was divided into three technical replicates. The dried lipid extracts were stored at −80 °C until further analysis.

For phospholipid analysis, dried lipid extracts were reconstituted in an LC-MS-grade solvent mixture of isopropanol, acetonitrile, and water at a ratio of 2:1:1. Deuterated lipid standards from Lipidomix were added as internal standards for estimating phospholipid concentrations. The samples were shaken at 20 Hz for 10 min and then incubated at room temperature for 30 min to ensure complete lipid dissolution. After centrifugation twice at 15,000× *g*, equal volumes from each sample were pooled to generate quality control (QC) samples.

For phospholipid quantification, 5.0 μL of each sample was injected, and one QC sample was injected after every 10 samples. Phospholipids were separated on a C18 column (ACQUITY UPLC BEH C18 column, 1.7 μm, 2.1 mm × 50 mm) and detected using a QTRAP 6500+ system (AB SCIEX) under negative ion mode with multiple reaction monitoring (MRM) transitions. The liquid chromatography conditions were as follows: Mobile phase A consisted of 33.3% acetonitrile, 33.3% methanol, 33.4% water, and 5.0 mM ammonium acetate; Mobile phase B consisted of 100% isopropanol containing 5.0 mM ammonium acetate. The flow rate was 0.15 mL/min. The gradient was set as follows: 0.0 min, 20% B; 1.0 min, 20% B; 3.0 min, 60% B; 20.0 min, 98% B; 20.1 min, 20% B; and 25 min, 20% B. Lipid species were identified by matching their chromatographic retention times and MRM transitions with the corresponding reference standards and the established lipidomics method. The detected MRM peaks were manually inspected to confirm peak assignment, and peak areas were quantified using Analyst OS v1.7.

For neutral lipid analysis, lipid extracts were reconstituted in an LC-MS-grade mixture of methanol and dichloromethane at a ratio of 1:1 containing 5.0 mM ammonium acetate, with Lipidomix used as the internal standard. A total of 5.0 μL of each sample was injected, and one QC sample was injected after every eight samples to ensure analytical consistency and reliability. TAGs and DAGs were separated using the same C18 column and detected in positive ion mode with MRM transitions.

### 2.6. Lipid Data Processing and Quantification

Lipid species were identified by matching their chromatographic retention times and MRM transitions with the corresponding reference standards. Quality-control samples were injected at regular intervals during the analytical sequence.

For each lipid species, the amount was estimated from the peak-area ratio between the analyte and its corresponding internal standard according to the following equation:

$$C_{s}=C_{std}\times \frac{A_{s}}{A_{std}}$$

where *C*_s_ is the estimated amount of the lipid species, *C*_std_ is the amount of the corresponding internal standard, *A*_s_ is the peak area of the analyte, and *A*_std_ is the peak area of the internal standard. For phospholipids, the internal-standard-corrected values were normalized to total protein content for total phospholipid quantification. The relative abundance of each phospholipid species was calculated as a percentage of the total phospholipid content, and phospholipid class composition was expressed as %. For neutral lipids, the internal-standard-corrected value was normalized to the corresponding total protein content.

### 2.7. Total RNA Was Isolated and Transcriptomic Analysis

Total RNA was isolated from primary mouse hepatocytes using RNA-easy Isolation Reagent (Cat. no. R701; Vazyme Biotech Co., Ltd., Nanjing, China.) following the manufacturer’s protocol. Transcriptomic analysis included n = 4 biological replicates per group per time point. After confirming RNA quality, libraries were constructed using the Illumina TruSeq RNA Preparation Kit. Sequencing was performed on Illumina HiSeq platforms with a 2 × 150 paired-end configuration at the Genewiz Core Facility. Low-quality sequences, including adapters and bases with a quality score below 20, were trimmed using Cutadapt (v1.9.1). Clean reads were aligned to the mouse reference genome (GRCm38) using Hisat2 (v2.2.1). Gene and isoform expression levels were quantified using HTSeq (v0.6.1). Differential expression analysis was performed using the DESeq2 Bioconductor package. The transcriptomic, lipidomic, metabolomic and WB datasets were obtained from the same independent pooled hepatocyte preparations.

### 2.8. Western Blotting

Cells were lysed in RIPA buffer (Beyotime, P0013B) supplemented with protease and phosphatase inhibitors (Beyotime, P1050 and P1051) and underwent ultrasonic lysis, followed by boiling at 75 °C for 10 min. Equal amounts of protein were separated by SDS-PAGE and transferred onto polyvinylidene difluoride (PVDF) membranes. After blocking with 5% milk for 1 h, membranes were incubated overnight at 4 °C with the following primary antibodies: PEMT (1:1000, Cat. no. ab172388; Abcam, Cambridge, UK), GAPDH (1:2000, Cat. no. CL594-60004; clone 1E6D9; Proteintech (Wuhan Sanying Biotechnology Co., Ltd.), Wuhan, China), FLAG (Sigma, Cat# F3165, 1:5000). The membranes were then incubated for 2 h at room temperature with the corresponding horseradish peroxidase-conjugated secondary antibodies, either anti-rabbit (CST, #7074, 1:5000, Danvers, MA, USA) or anti-goat (Abcam, ab97110, 1:5000). Protein signals were detected using the Super ECL Chemiluminescence Kit (Vazyme, E411).

### 2.9. Statistical Analysis

Data are presented as mean ± standard deviation. Biological replicate numbers are provided in the figure legends. Hepatocytes isolated from 2 mice were pooled before plating. Four wells from each pooled cell suspension were used as replicates. Statistical analyses were performed using GraphPad Prism 9 (GraphPad Prism version 9; GraphPad Software, Inc., San Diego, CA, USA) and R version4.4.2 (R Foundation for Statistical Computing, Vienna, Austria). Two-group comparisons at the same time point were performed using two-tailed unpaired Student’s *t*-tests. Comparisons involving multiple time points or experimental factors were analyzed by two-way ANOVA with culture time, genotype or treatment as fixed factors, followed by multiple-comparison correction. For lipidomic analysis, raw peak areas were extracted using Analyst OS and processed according to our laboratory-established lipidomics workflow [25,26]. Statistical significance was defined as * *p* < 0.05, ** *p* < 0.01 vs. controls. During sample extraction, a parallel aliquot from each sample was retained for protein quantification. Total protein content was measured using the BCA assay, and metabolite or lipid signals were normalized to the corresponding protein amount.

## 3. Results

### 3.1. Time-Dependent Phospholipid Homeostasis Remodeling and Suppression of De Novo Phospholipid Synthesis During In Vitro Hepatocyte Culture

**Figure 1:**
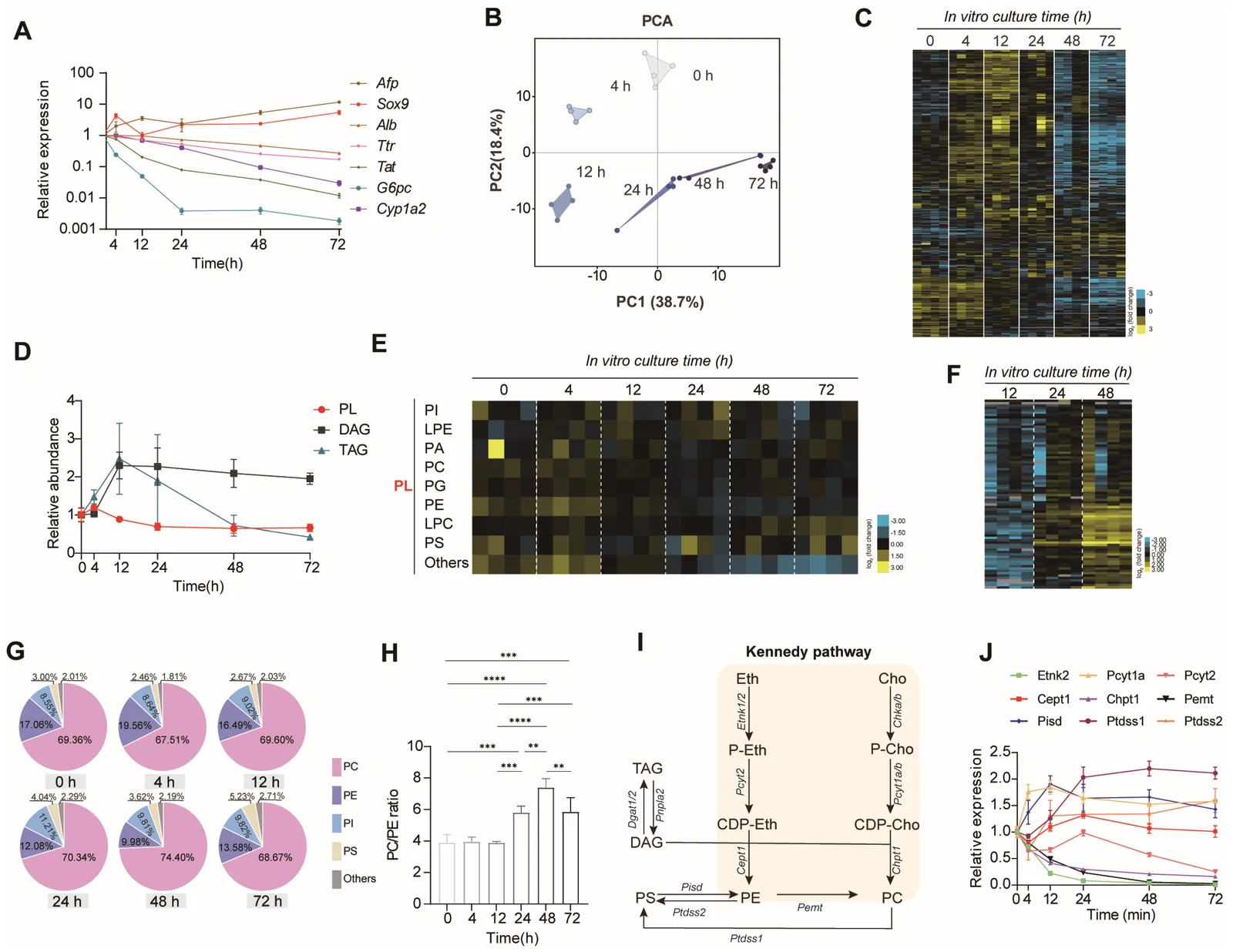
Time-dependent phospholipid remodeling and suppression of phospholipid synthesis pathways during primary mouse hepatocyte culture. (**A**) Expression changes of hepatocyte functional genes and hepatic progenitor marker genes during culture. (**B**) Principal component analysis of lipidomic profiles across the culture time course. (**C**) Heatmap showing time-dependent remodeling of lipid species. (**D**) Total levels of major lipid classes, including phospholipids (PL), phosphatidylcholine (PC), phosphatidylethanolamine (PE), diacylglycerol (DAG) and triacylglycerol (TAG). (**E**) Temporal changes in PL, PC, PE, etc., levels in WT cells. (**F**) Heat map of phospholipid changes in the culture medium. (**G**), Relative composition of major phospholipid classes during culture. (**H**) PC/PE ratio during 0–72 h of culture. (**I**) Schematic or expression overview of genes involved in PE and PC synthesis pathways. (**J**) Expression changes of key Kennedy pathway and PEMT pathway genes, including *Etnk2*, *Pcyt2*, *Pcyt1a*, *Cept1*, *Chpt1* and *Pemt*, etc. Data are presented as mean ± SEM. n = 4 replicate wells from a single pooled hepatocyte preparation generated by pooling cells from 2 mice. Statistical significance was determined as described in Methods. ** *p* < 0.01, *** *p* < 0.001, **** *p* < 0.0001.

Hepatic function genes declined progressively with culture time, whereas hepatic progenitor marker genes increased (Figure 1A), consistent with dedifferentiation and loss of hepatocyte function in conventional culture [4,27].

Using lipidomics, we observed marked time-dependent lipidome remodeling in primary mouse hepatocytes during 0–72 h of culture [1]. PCA and heatmap analyses showed progressive separation by culture time (Figure 1B,C). Total phospholipids (PL), including PC and PE, decreased over time, the LPC and LPE increased over time, whereas DAG and TAG peaked at 24 h, with TAG declining thereafter and DAG remaining relatively stable (Figure 1D,E); the phospholipids in the 12, 24, and 48 h culture media were continuously accumulating (Figure 1F). Phospholipid composition changed differentially over culture time rather than decreasing proportionally (Figure 1G). The PC/PE ratio remained stable from 0 to 12 h, increased at 24 h, and peaked at 48 h (Figure 1H).

PC and PE are mainly synthesized through the Kennedy pathway [28], gene expression analysis of the phospholipid synthesis network showed restriction of key Kennedy pathway steps during culture (Figure 1I). In the PE branch, *Etnk2* rapidly decreased and *Pcyt2* declined at later stages, whereas in the PC branch, *Chpt1* and *Pemt* were progressively downregulated despite stable or increased *Pcyt1a* expression (Figure 1J). These results indicate that the expression of genes involved in de novo PE/PC synthesis was altered during primary hepatocyte culture, accompanied by a sustained decline in the *Pemt* expression PE-to-PC pathway [21,29]. Thus, the decline in *Pemt* expression occurred within the broader context of altered PC/PE balance and membrane phospholipid remodeling.

### 3.2. PEMT Knockout Does Not Aggravate Hepatocyte Dedifferentiation but Rewires Culture-Triggered Lipidomic Trajectories

**Figure 2:**
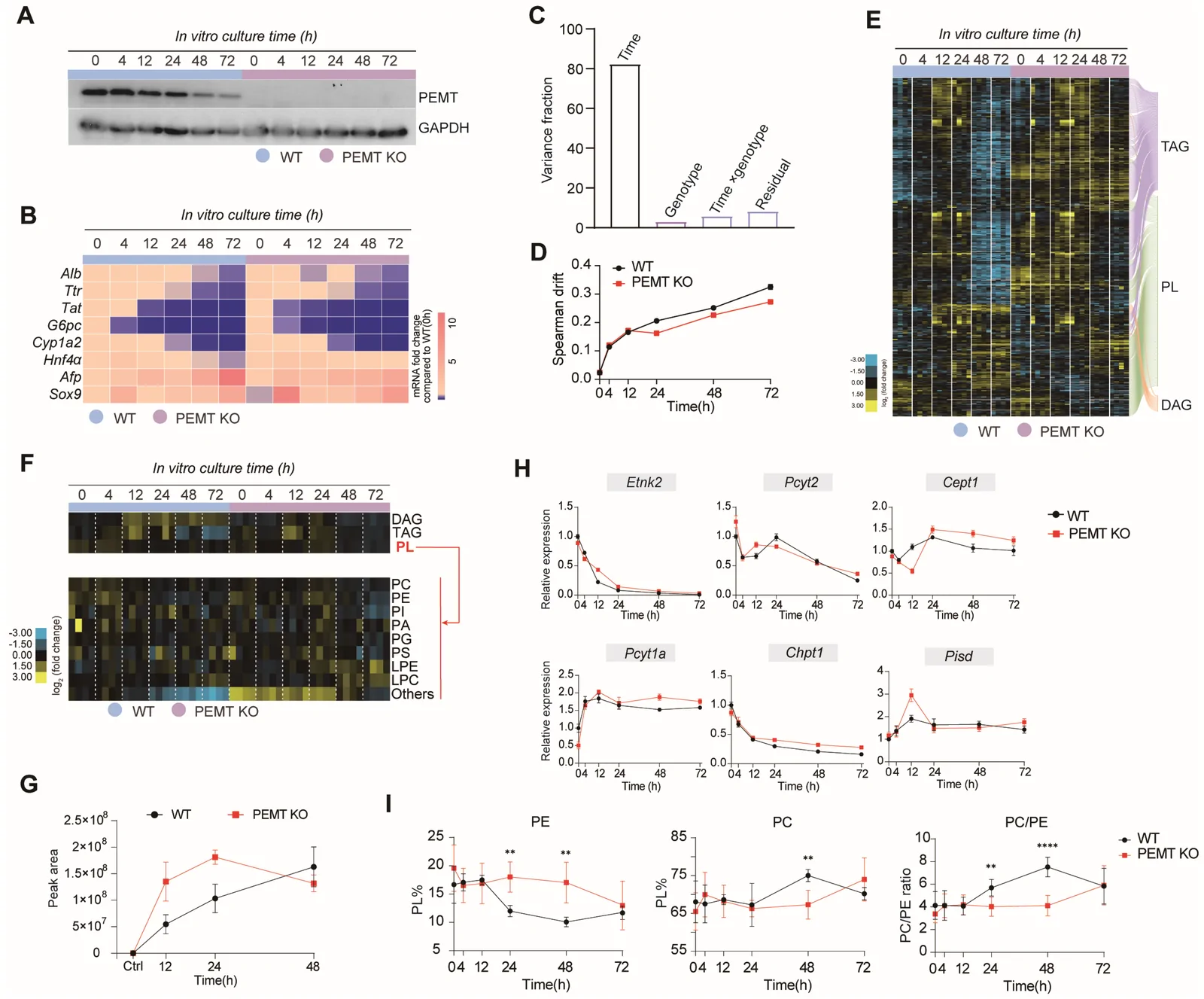
PEMT knockout does not markedly aggravate dedifferentiation but alters culture-associated transcriptomic and lipidomic trajectories. (**A**) PEMT expression in WT primary hepatocytes during culture and validation of PEMT loss in KO hepatocytes. (**B**) Expression patterns of hepatocyte functional genes and hepatic progenitor marker genes in WT and PEMT KO cells. (**C**) Variance decomposition of transcriptomic profiles showing the relative contribution of culture time, genotype and the time-by-genotype interaction to global transcriptomic variation. (**D**) Global transcriptomic deviation score calculated relative to the genotype-matched 0 h reference state. (**E**) Heatmap analysis of lipidomic profiles in WT and PEMT KO hepatocytes during culture. (**F**) Temporal changes in PL, PC, PE, DAG and TAG levels in WT and PEMT KO cells. (**G**) The changes in total phospholipids in the cell culture medium of the WT and PEMT KO (Ctrl: cell-free incubation medium). (**H**) Expression changes of Kennedy pathway genes involved in PE and PC synthesis. (**I**) Relative PE and PC abundance and PC/PE ratio in WT and PEMT KO hepatocytes. Data are presented as mean ± SEM. Sample analysis included n = 4 biological replicates per group per time point. For line plots comparing two groups over time, asterisks indicate significant differences between the two groups at the same time point. ** *p* < 0.01; **** *p* < 0.0001.

Given the continuous decline of *Pemt* expression in WT primary hepatocytes during culture, PEMT KO hepatocytes were used to determine whether PEMT loss altered culture-associated membrane phospholipid remodeling (Figure 2A). WT and PEMT KO cells showed similar time-dependent changes in hepatic function genes and hepatic progenitor markers, indicating comparable culture-induced dedifferentiation (Figure 2B). Variance decomposition [30] showed that culture time explained 82.46% of the total transcriptomic variation, whereas genotype and the time-by-genotype interaction explained 3.19% and 5.95%, respectively (Figure 2C). Global transcriptomic deviation [31] increased progressively in both groups and diverged after 24 h, suggesting that PEMT deficiency modified the trajectory of culture-associated transcriptional changes without overriding the dominant effect of culture duration [27,32] (Figure 2D). We next asked whether these PEMT-dependent transcriptional differences were accompanied by corresponding changes in lipid composition.

Heatmap analyses showed progressive lipidome remodeling in both WT and PEMT KO cells, with incomplete overlap between genotypes at the same time (Figure 2E). PL, PC, and PE decreased in both groups, indicating a dominant culture–time effect, the LPC and LPE are showing an upward trend, whereas PE, PC, and DAG showed genotype-dependent temporal patterns (Figure 2F). Following the replacement of plating medium with incubation medium at 4 h, phospholipid levels in the medium were accumulated at 12, 24, and 48 h (Figure 2G).

WT and PEMT KO cells showed similar transcriptional changes in Kennedy pathway genes, including reduced *Etnk2* and *Pcyt2* in the PE branch and sustained *Chpt1* downregulation in the PC branch (Figure 2H). Despite this shared restriction of de novo phospholipid synthesis, PEMT KO cells retained more PE, showed a greater PC decrease, and had a limited increase in the PC/PE ratio (Figure 2I). This indicates that the main role of PEMT deficiency did not markedly alter the de novo PE/PC synthesis gene expression, but was associated with the PC/PE compositional balance and lipid remodeling trajectory.

### 3.3. PEMT Deficiency Selectively Remodels Molecular Species, Acyl Chain Length and Unsaturation Profiles of PC and PE

**Figure 3:**
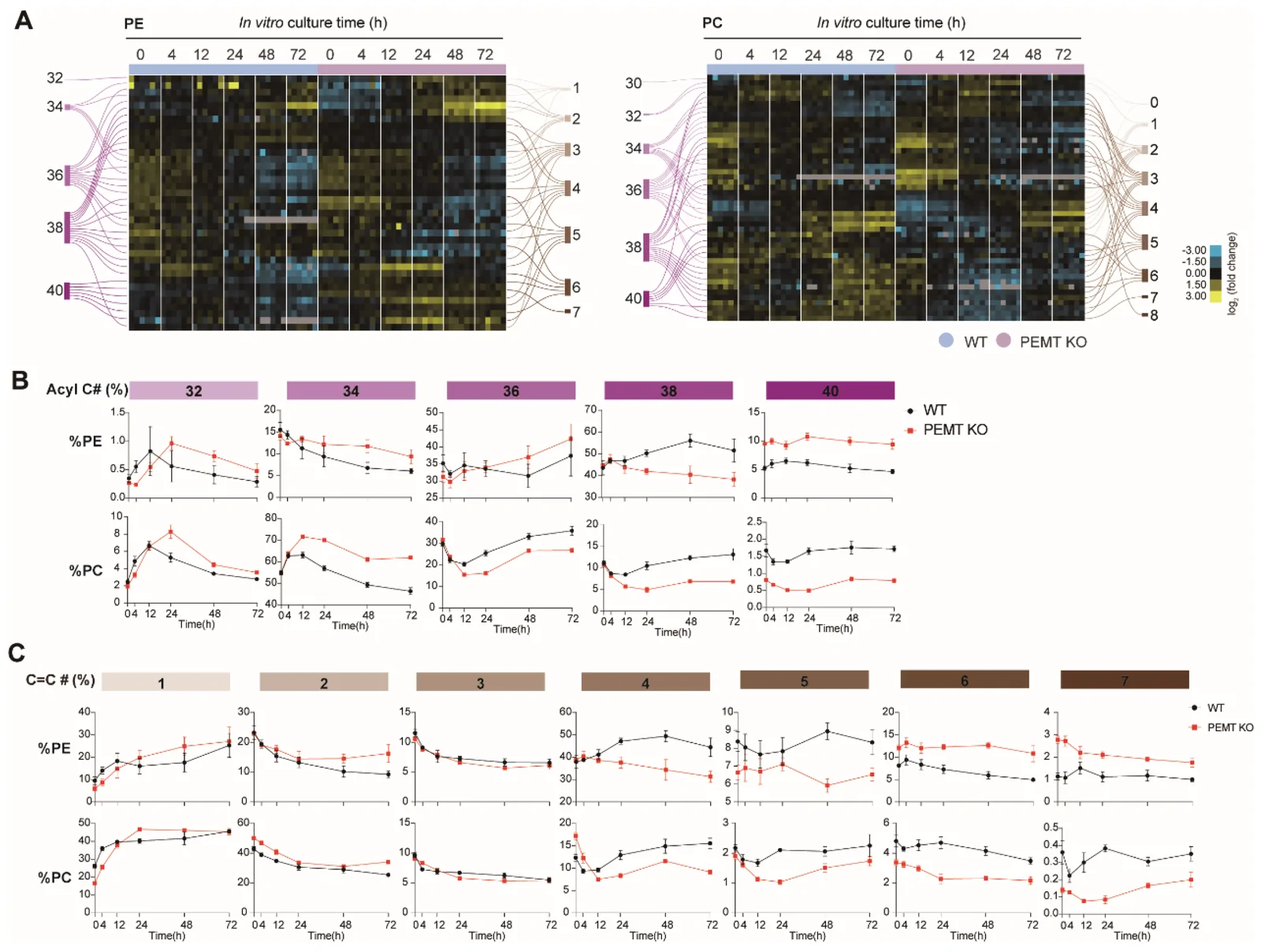
PEMT deficiency selectively remodels PE and PC molecular species, acyl-chain length and unsaturation profiles. (**A**) Heatmaps showing molecular species-level remodeling of PE and PC in WT and PEMT KO cells. (**B**) Relative distribution of PE and PC species grouped by total acyl-chain carbon number. PEMT deficiency reduced the accumulation of 38C PE, maintained a higher proportion of 40C PE, retained 34C PC and limited the later increase in 36C and 38C PC. (**C**) Relative distribution of PE and PC species grouped by total double-bond number. PEMT deficiency reduced 4-DB PE enrichment, retained more highly unsaturated PE species and limited the maintenance of moderately to highly unsaturated PC species during culture. Data are presented as mean ± SEM. Sample analysis included n = 4 replicate wells from a single pooled hepatocyte preparation generated by pooling cells from 2 mice.

PEMT deficiency did not markedly alter de novo PE and PC synthesis gene expression but changed the PC/PE balance. We therefore analyzed PE and PC molecular species, acyl-chain length, and unsaturation to assess phospholipid structural remodeling [33]. Phospholipid molecular-level heatmaps and structural stratification showed time-dependent remodeling of PE and PC species in both WT and PEMT KO cells, with genotype-dependent differences in specific species (Figure 3A).

Classification by total acyl-chain carbon number showed chain-length-specific changes in PE and PC composition during culture (Figure 3B). In PE, 38C species were the dominant fraction. They increased in WT cells from approximately 43% at 0 h to 56% at 48 h and remained at 51% at 72 h, whereas in PEMT KO cells they decreased to approximately 38% at 72 h after a transient increase at 4 h. In contrast, 40C PE was maintained at approximately 10% in PEMT KO cells, compared with approximately 5% in WT cells. For PC, 34C species were the dominant fraction. In PEMT KO cells, 34C PC peaked at approximately 71% at 12 h and remained at approximately 61% at 48–72 h, higher than in WT cells at the same stages. Conversely, 36C and 38C PC decreased initially and then increased in both groups, but remained lower in PEMT KO cells. These data show that PEMT deficiency reduced 38C PE accumulation, maintained higher 40C PE, retained 34C PC, and limited the later increase in 36C and 38C PC during culture.

Classification by total double-bond number showed unsaturation-specific changes in PE and PC during culture [33] (Figure 3C). In PE, four-double-bond (DB) species were the dominant fraction. They increased in WT cells from approximately 38% at 0 h to 49% at 48 h, whereas in PEMT KO cells, they decreased from approximately 39% at 0 h to 31% at 72 h. In contrast, 6-DB and 7-DB PE remained higher in PEMT KO cells than in WT cells, while 2-DB PE decreased less markedly in PEMT KO cells. For PC, 1-DB species increased in both groups and exceeded 40% at later stages, whereas 2-DB species decreased. PEMT KO cells showed lower 4–6-DB PC levels than WT cells, with 4-DB PC decreasing rapidly at early stages and showing limited recovery. These data show that PEMT deficiency reduced 4-DB PE enrichment, retained highly unsaturated PE species, and limited the maintenance of moderately to highly unsaturated PC species during culture.

### 3.4. Exogenous PEMT Expression Is Associated with PC/PE Balance but Has Limited Effects on Molecular Structural Remodeling

**Figure 4:**
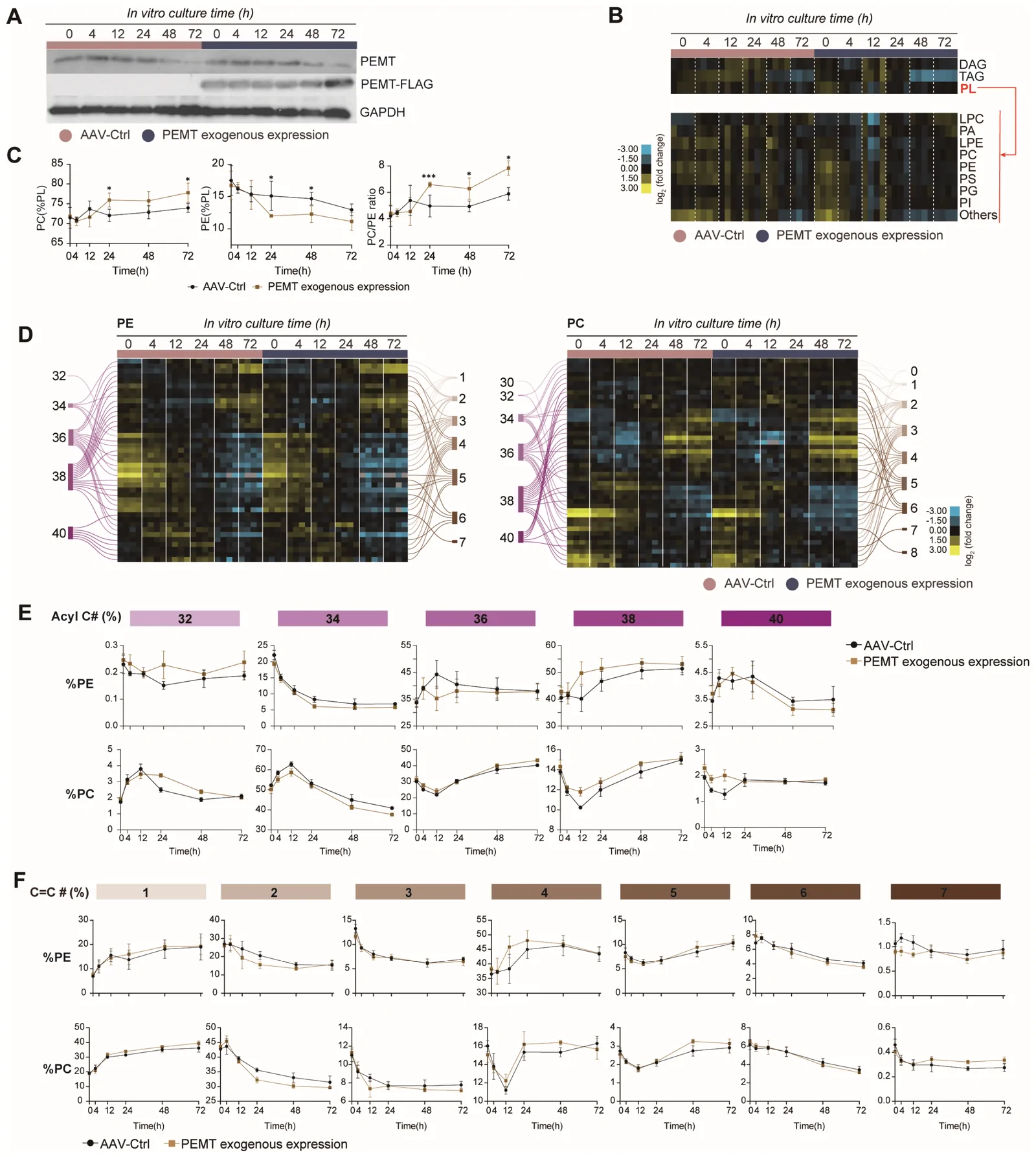
Expression of exogenous PEMT redirects PC/PE balance but has limited effects on phospholipid structural remodeling. (**A**) Expression of exogenous FLAG-tagged PEMT was detected by anti-FLAG Western blotting. (**B**) Temporal changes in major lipid classes, including PL, PC, PE, DAG and TAG, in control and PEMT exogenous expression cells. (**C**) Relative PC and PE proportions and PC/PE ratio during culture. (**D**) Heatmaps showing PE and PC molecular species profiles in control and PEMT exogenous expression cells. (**E**) Relative distribution of PE and PC species grouped by total acyl-chain carbon number. (**F**) Relative distribution of PE and PC species grouped by total double-bond number. PEMT overexpression increased PC proportion and PC/PE ratio but did not fully restore the broader molecular structural trajectory imposed by culture duration. Data are presented as mean ± SEM. Sample analysis included n = 4 replicate wells from a single pooled hepatocyte preparation generated by pooling cells from 2 mice. For line plots comparing two groups over time, asterisks indicate significant differences between the two groups at the same time point. * *p* < 0.05; *** *p* < 0.001.

Because *Pemt* expression declined during culture and PEMT deficiency redirected PC/PE balance, we next used PEMT exogenous expression model as a gain-of-function test to determine whether PEMT exogenous expression could redirect culture-associated phospholipid remodeling [34]. Western blot analysis using an anti-FLAG antibody detected a clear FLAG-tagged PEMT signal in the PEMT exogenous expression group, whereas no corresponding signal was observed in the control group, confirming AAV-mediated expression of FLAG-tagged PEMT. However, the overall PEMT signal showed a time-dependent pattern similar to that observed in AAV-Ctrl cells, without a sustained increase at 48 and 72 h (Figure 4A).

Analysis of major lipid classes showed that PL, PC, and PE decreased overall in both groups, whereas DAG and TAG increased initially and then declined (Figure 4B). At the phospholipid class level, PEMT exogenous expression cells showed a higher PC proportion, a lower PE proportion, and an increased PC/PE ratio from 24 to 72 h (Figure 4C). Thus, PEMT exogenous expression did not eliminate the dominant culture-time effect, but was associated with a partial shift in the culture-associated PC/PE balance toward PC enrichment. Molecular species heatmaps further showed that PE and PC profiles remained primarily organized by culture time, with comparatively modest PEMT-dependent shifts after PEMT exogenous expression (Figure 4D).

Acyl-chain length analysis showed that both PE and PC underwent pronounced chain length-selective remodeling during in vitro culture (Figure 4E). For PE, 38C and 36C remained the two most abundant acyl-chain length groups, and PEMT exogenous expression did not alter the overall compositional framework. Both groups showed similar time-dependent trends. For PC, 34C and 36C were the predominant acyl-chain length groups. Both the control and PEMT exogenous expression groups showed a gradual decrease in 34C PC and a progressive increase in 36C and 38C PC during culture; however, no significant differences were observed between the two groups.

Analysis of total double-bond number showed that both PE and PC underwent pronounced time-dependent remodeling during in vitro culture (Figure 4F). PE was dominated by species with four double bonds. Both the control and PEMT exogenous expression groups showed a gradual increase in 4-DB PE, accompanied by decreases in 2-DB and 3-DB PE. PC was mainly composed of species with one or two double bonds. During culture, 1-DB PC gradually increased, whereas 2-DB and 3-DB PC decreased; meanwhile, 4-DB and 5-DB PC showed partial recovery at the middle-to-late culture stages. Compared with the control group, PEMT exogenous expression did not alter the overall direction of these changes.

Together, these data show that PEMT exogenous expression produced a class-level gain-of-function effect opposite to PEMT deficiency, increasing PC proportion and the PC/PE ratio while a lower PE proportion. However, PEMT exogenous expression only modestly affected PE and PC molecular species, acyl-chain length, and unsaturation distribution. Thus, PEMT exogenous expression was associated with a partial shift in culture-associated PC/PE balance, but insufficient to fully reset the broader molecular structural trajectory imposed by in vitro culture.

### 3.5. PEMT Perturbation Differentially Alters Methionine–SAM–SAH Metabolic Trajectories

**Figure 5:**
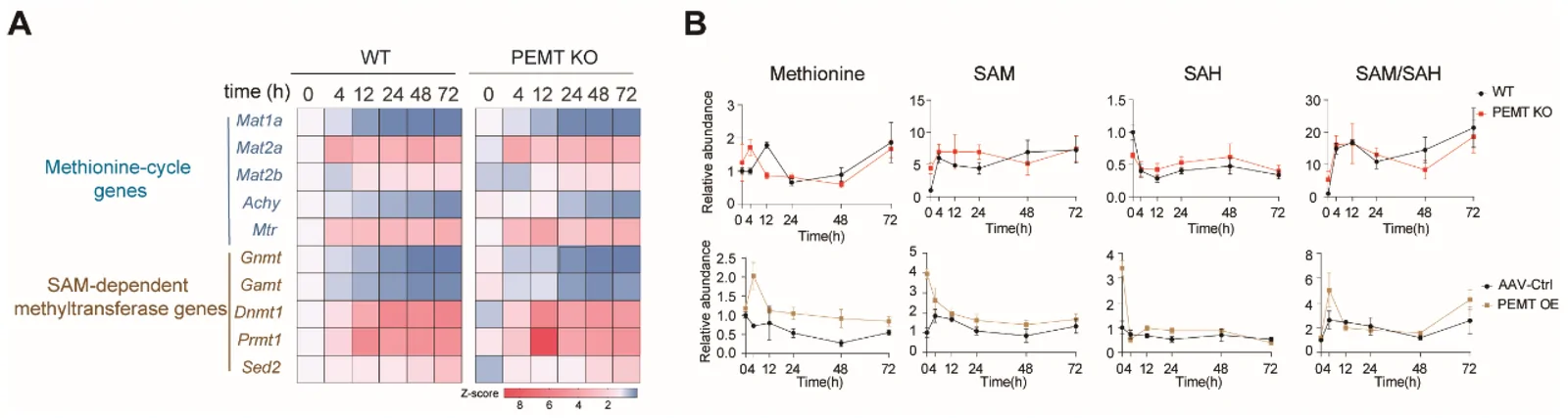
PEMT perturbation is associated with dynamic remodeling of the methionine–SAM–SAH axis. (**A**) Expression patterns of genes related to methionine–SAM–SAH metabolism in WT and PEMT KO hepatocytes, including *Mat1a*, *Mat2a*, *Mat2b*, *Gnmt*, *Gamt*, *Dnmt1*, *Prmt1* and *Setd2*. (**B**) Targeted metabolomic analysis of methionine, SAM and SAH levels in PEMT KO and PEMT exogenous expression models during culture. PEMT KO and PEMT exogenous expression altered basal levels and temporal trajectories of methyl-donor metabolites, but their effects were not simply reciprocal. Data are presented as mean ± SEM. Sample analysis included n = 4 replicate wells from a single pooled hepatocyte preparation generated by pooling cells from 2 mice.

Because PEMT-mediated conversion of PE to PC consumes SAM and generates SAH [35,36], we next examined whether PEMT perturbation altered the methionine–SAM–SAH axis during hepatocyte culture. Analysis of genes related to the methionine–SAM–SAH axis in the transcriptomic dataset revealed similar temporal expression patterns in WT and PEMT KO hepatocytes (Figure 5A). *Mat1a* expression decreased during culture, whereas *Mat2a* and *Mat2b* increased. *Gnmt* and *Gamt* also decreased, while *Dnmt1*, *Prmt1*, and *Setd2* increased. These temporal patterns were largely preserved in PEMT KO cells, indicating that PEMT deficiency did not markedly alter the culture-associated expression changes of these selected genes [37].

The targeted metabolomics analysis revealed the levels of methionine, SAM and SAH within the cells. (Figure 5B). In the PEMT KO model, methionine varied over time without a consistent genotype-dependent direction. SAM increased by 4 h and remained elevated in both groups, but was higher in KO than WT cells at 0 h. At the same baseline, SAH in KO cells was approximately 50% of the WT level, resulting in a higher SAM/SAH ratio. In the PEMT exogenous expression model, methionine was higher in OE cells than in controls from 24 to 72 h. At 0 h, both SAM and SAH were higher in OE cells, with SAH reaching approximately three- to fourfold the control level. Both metabolites declined rapidly after plating. SAM stabilized from 12 to 72 h, whereas SAH approached control levels by 4 h. Overall, both PEMT KO and PEMT exogenous expression were associated with altered basal levels and dynamic trajectories of the methionine–SAM–SAH axis, but their effects were not simply opposite to each other.

### 3.6. Methionine Deprivation Remodels PC/PE Balance and Phospholipid Structural Composition

**Figure 6:**
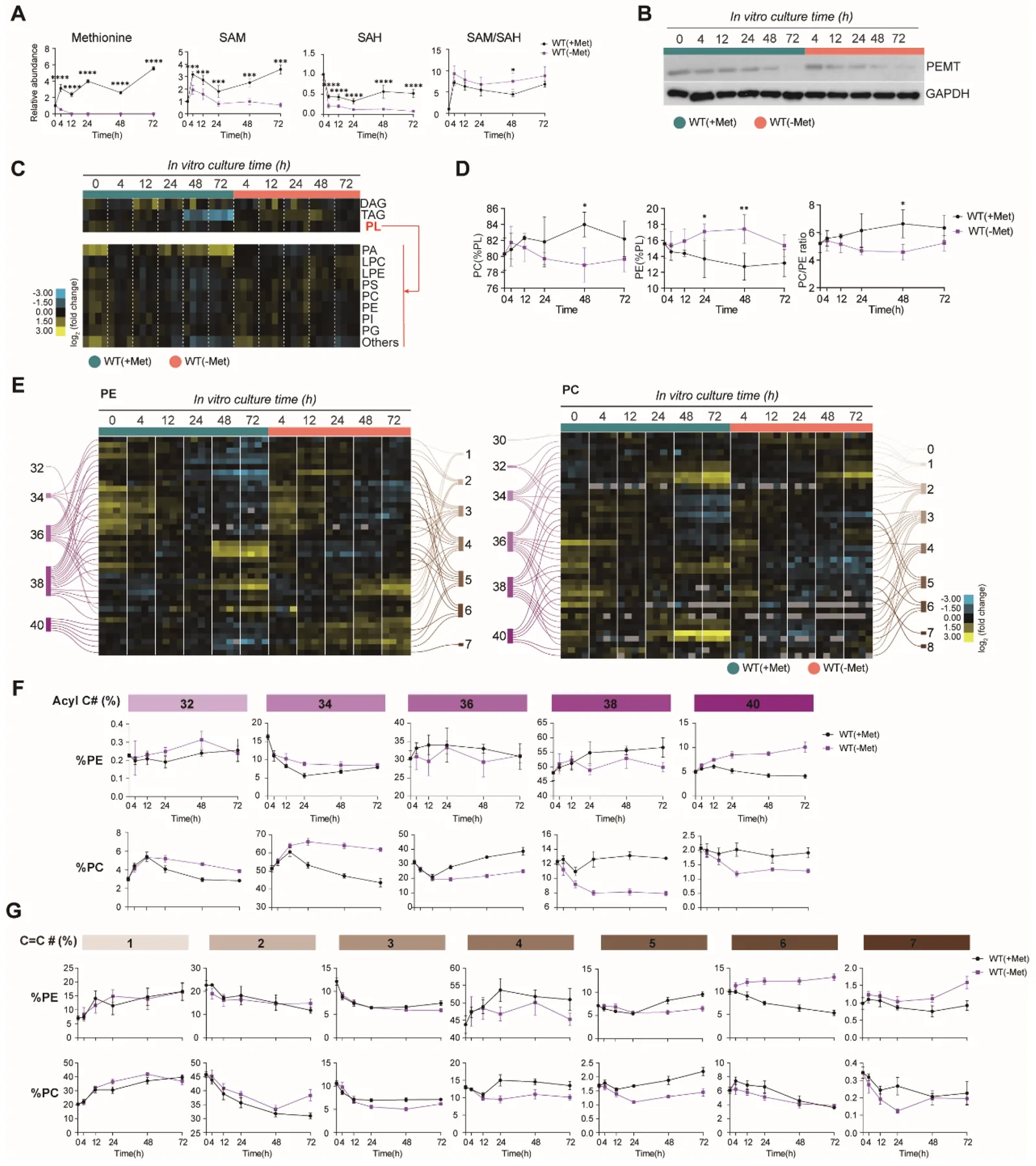
Methionine deprivation remodels PC/PE balance and phospholipid structural composition in primary mouse hepatocytes. (**A**) Intracellular methionine, SAM and SAH levels confirming effective perturbation of the methionine–SAM–SAH axis. (**B**) PEMT protein expression under +Met and -Met conditions during culture. (**C**) Temporal changes in PL, PC, PE, DAG and TAG levels under +Met and -Met conditions. (**D**) Relative PC and PE proportions and PC/PE ratio, showing PE retention and reduced PC/PE ratio under methionine deprivation during the middle-to-late culture period. (**E**) Heatmaps showing time-dependent remodeling of PE and PC molecular species under +Met and -Met conditions. (**F**) Relative distribution of PE and PC species grouped by total acyl-chain carbon number. Methionine deprivation attenuated 38C PE accumulation, increased 40C PE and retained more 34C PC at later stages. (**G**) Relative distribution of PE and PC species grouped by total double-bond number. Methionine deprivation reduced 4-DB PE enrichment, maintained higher 6-DB and 7-DB PE proportions and limited the recovery of moderately unsaturated PC species. Data are presented as mean ± SEM. Sample analysis included n = 4 replicate wells from a single pooled hepatocyte preparation generated by pooling cells from 2 mice. For line plots comparing two groups over time, asterisks indicate significant differences between the two groups at the same time point. * *p* < 0.05; ** *p* < 0.01; *** *p* < 0.001; **** *p* < 0.0001.

Because methionine is linked to methyl-donor metabolism, we next tested whether methionine deprivation was associated with changes in PC/PE remodeling. Removal of methionine from the culture medium reduced intracellular methionine, SAM, and SAH, confirming effective perturbation of the methionine–SAM–SAH axis (Figure 6A). PEMT protein showed a similar time-dependent pattern under methionine-replete and methionine-deprived conditions, indicating that methionine deprivation did not alter its overall expression trajectory (Figure 6B).

PL, PC, and PE decreased with culture time in both WT(+Met) and WT(-Met) cells, indicating that methionine deprivation did not prevent the overall loss of phospholipids (Figure 6C). DAG peaked at 12 h in both conditions and then declined. TAG increased transiently before declining in WT(+Met) cells, but decreased overall in WT(-Met) cells. Despite the shared phospholipid loss, WT(-Met) cells had a lower PC proportion at 48 h and a higher PE proportion at 24–48 h. Consequently, the PC/PE ratio was lower in WT(-Met) cells during the 24–48 h period (Figure 6D). Thus, methionine deprivation redirected the class-level PC/PE balance toward PE retention during the middle-to-late culture period. The heatmaps showed time-dependent remodeling of PE and PC species. WT(-Met) cells displayed distinct patterns from WT(+Met) at matched time points, indicating that methionine deprivation redirected PE and PC molecular remodeling rather than uniformly reducing phospholipid abundance (Figure 6E).

Acyl-chain length analysis showed that both PE and PC underwent pronounced chain length-selective remodeling during culture (Figure 6F). For PE, 38C and 36C remained the two most abundant acyl-chain groups in both conditions. In WT(+Met) cells, 38C PE increased from 12 to 72 h, whereas this increase was attenuated in WT(-Met) cells. In contrast, 40C PE increased progressively in WT(-Met) cells but remained lower in WT(+Met) cells. For PC, 34C and 36C were the predominant groups. In both conditions, 34C PC increased during early culture, whereas 36C and 38C PC initially decreased. At later stages, WT(-Met) cells retained more 34C PC but showed limited recovery of 36C and 38C PC compared with WT(+Met) cells.

Analysis of total double-bond number showed that PE and PC underwent unsaturation-selective remodeling under both conditions (Figure 6G). For PE, 4-DB species remained the dominant fraction. In WT(+Met) cells, 4-DB PE increased to a peak at 24 h and remained above its initial level, whereas it was generally lower in WT(-Met) cells from 12 to 72 h and declined at later stages. Conversely, 6-DB and 7-DB PE were maintained at higher proportions in WT(-Met) cells, while 5-DB PE was lower at later stages. For PC, 1-DB and 2-DB species were the predominant fractions. The proportion of 1-DB PC increased in both conditions, whereas 2-DB PC decreased before a partial recovery at 72 h in WT(-Met) cells. WT(-Met) cells also showed generally lower proportions of 3-DB to 5-DB PC, particularly 4-DB and 5-DB species at later stages. Overall, methionine deprivation had divergent effects on the structural remodeling of PE and PC, showing clear phospholipid class specificity.

## 4. Discussion

The main finding of this study is that primary mouse hepatocytes undergo a time-dependent process of membrane phospholipid reorganization during in vitro culture. Although phospholipids decreased overall, the levels of different phospholipid classes did not decline synchronously or proportionally. The medium total phospholipids in the culture medium accumulated from 12 to 48 h, showing an opposite change to the decline observed in cells. Instead, the PC/PE balance, the molecular species of PE and PC, acyl chain lengths, and the distribution of unsaturation exhibited sustained and distinct patterns of change. This reorganization process correlates with the gradual loss of hepatocyte identity and a decline in PEMT expression levels. PEMT gene deletion shifts the PC/PE balance toward PE retention and alters the trends in phospholipid structural changes, but does not significantly enhance the overall pattern of dedifferentiation markers. Conversely, PEMT overexpression shifts the balance of phospholipid categories toward PC, but has a limited restorative effect on structure. Methionine deprivation reduced the levels of methionine, SAM, and SAH, and produced lipid changes that partially overlapped with those observed in PEMT-knockout hepatocytes. In summary, these results redefine culture-associated lipidomic changes as an ordered process of membrane phospholipid rearrangement, the direction of which is selectively determined by PEMT status and methionine availability.

These findings suggest that, during cell culture, a reduction in phospholipids, transcriptional changes in specific synthetic genes, and structural redistribution constitute a broad background, while PEMT status was associated with differences in PC/PE composition within this broader remodeling program. This is consistent with the observation that was associated with differences in the PC/PE balance and molecular structure without significantly accelerating dedifferentiation. This is also consistent with the observation that PEMT exogenous expression was associated with the differences in lipid class composition but had limited effects on the broader phospholipid structural trajectory. Changes in acyl-chain length and unsaturation provide structural information beyond total PC, PE and PC/PE ratio. These features influence membrane packing, curvature, fluidity and membrane-associated protein organization [15,33,38,39]. Thus, the time-dependent redistribution of PE and PC species suggests that culture-induced phospholipid remodeling involves selective changes in membrane physical properties, rather than phospholipid loss alone [16,40,41]. The retention of longer-chain and more unsaturated PE species may be consistent with differences in substrate availability or acyl-chain remodeling, whereas the accumulation of shorter-chain or less unsaturated PC species may indicate a greater contribution from Kennedy pathway-derived PC or acyl-chain remodeling [42,43]. Importantly, PEMT deficiency and methionine deprivation selectively changed the chain-length and unsaturation profiles of PE and PC, consistent with the possibility that PEMT status and methyl-donor availability are associated with differences in the phospholipid structural remodeling of phospholipid remodeling beyond class-level PC/PE balance.

Methionine, SAM and SAH profiles in PEMT KO and PEMT exogenous expression cells further indicate that a simple substrate-product model cannot fully explain these changes. A broader metabolic link between phospholipids and histone methylation has been demonstrated in cellular systems [22,44]. However, steady-state metabolite concentrations reflect simultaneous production and consumption, whereas the KO and OE models differ in their developmental history and the timing of intervention. The results of methionine deprivation are associated with membrane phospholipid remodeling and methionine metabolism. Methionine deprivation may affect PEMT-dependent PE-to-PC conversion by limiting SAM availability, but its lipid effects cannot be attributed specifically to PEMT because it has broader metabolic effects.

This explanation is partly consistent with previous studies, while also expanding upon them. Primary hepatocytes undergo major metabolic changes immediately after isolation, and these changes persist or intensify during culture [6,27,41]. Lipidomic studies further indicate that hepatocyte dedifferentiation is accompanied by changes in the molecular lipid profile, including a decrease in polyunsaturated fatty acids and an increase in saturated or monounsaturated fatty acids [13]. Our findings support the reorganization of lipid metabolism during the loss of hepatocyte identity, but go further to reveal the dynamic trajectories of the PC/PE ratio [33,45], chain length, and unsaturation levels over the 0–72 h period. Previous studies have also confirmed that a reduced PC/PE ratio in the liver impairs cell membrane integrity and promotes the development of steatohepatitis [16]. Although this study did not identify comparable membrane damage, it established that PC/PE remodeling is a measurable feature of the adaptation process in culture. Therefore, the innovation lies not in assigning a fixed acyl chain preference to PEMT, but in revealing the time-resolved trajectories of phospholipids in hepatocytes undergoing metabolic changes as influenced by PEMT status.

The significance of these findings lies in the fact that they provide a more precise framework for understanding and monitoring the adaptation of primary hepatocytes during culture. Conceptually, this study goes beyond the scope of total lipid loss and identifies membrane phospholipid composition as a dynamic component of culture-related state transitions., The study identifies PEMT status as being associated with PC/PE rearrangement and suggests that methionine availability may contribute to these differences. In practical applications, the PC/PE ratio, as well as the chain length and unsaturation distributions of PE and PC, can complement traditional hepatocyte markers when defining culture stages or comparing culture conditions [46,47]. Nevertheless, this study has several limitations, first, as PEMT activity, the flux from PE to PC, membrane-specific lipids, and functional consequences were not directly measured [48,49]. Second, because the 0 h samples were collected before plating whereas later samples were adherent cells exposed to culture medium, early differences may also reflect cell attachment, medium transition, and selection of adherent cells. Third, extracellular phospholipid accumulation does not establish active phospholipid efflux, because contributions from cell death, membrane shedding, extracellular vesicles, and cellular debris cannot be excluded. Fourth, exogenous FLAG-tagged PEMT was detectable, the transduction efficiency and percentage of GFP-positive hepatocytes were not quantitatively assessed. Because Western blotting was performed on bulk lysates, these data do not establish a sustained increase in total PEMT across the entire hepatocyte population. Fifth, methionine deprivation affects multiple PEMT-independent processes, and the absence of factorial and rescue experiments prevents us from assigning its lipid effects specifically to PEMT. Finally, although EGFP was included in both AAV vectors, the proportion of GFP-positive cells and GFP-specific toxicity was not independently assessed. Whether primary human hepatocytes undergo a similar rearrangement program remains a key unresolved question. Within these constraints, the study describes a time-resolved membrane lipid trajectory associated with culture duration, PEMT status, and methionine availability.

## 5. Conclusions

In conclusion, primary mouse hepatocytes undergo time-dependent membrane phospholipid remodeling during 0–72 h of in vitro culture. This process involves non-proportional changes in PC/PE balance and continuous redistribution of PE and PC molecular structures. PEMT perturbation was associated with PEMT selective differences in phospholipid composition but did not broadly determine culture-induced dedifferentiation. Methionine deprivation and the associated changes in SAM and SAH were consistent with an association between methionine-related metabolism and phospholipid remodeling, although the present measurements do not establish a PEMT-specific mechanism, PE-to-PC flux, or methylation flux. Together, these findings describe a time-resolved membrane lipid trajectory associated with hepatocyte adaptation to culture, PEMT status, and methionine availability, while its functional consequences and relevance to human hepatocytes remain to be established.

## Data Availability

The data supporting reported results are available on request from the corresponding author (A.C.).
